# Towards a shared service centre for telemedicine: Telemedicine in Denmark, and a possible way forward

**DOI:** 10.1177/1460458215592042

**Published:** 2015-08-10

**Authors:** Simon Bo Larsen, Nanna Skovgaard Sørensen, Matilde Grøndahl Petersen, Gitte Friis Kjeldsen

**Affiliations:** The Alexandra Institute Ltd, Denmark; Centre for Telemedicine and Telehealthcare, Central Denmark Region, Denmark; MedTech Innovation Consortium, Denmark

**Keywords:** organisation, participatory design, process of change, service centre, support, telecare, telehealth, telemedicine

## Abstract

Although evidence of the effectiveness of telemedicine is accumulating, knowledge of *how* to make best use of telemedicine is limited. This article presents results from a multi-stakeholder project that developed a new concept, a ‘shared service centre’ for telemedicine that is envisioned as working across different telemedical initiatives to support the implementation and wider adoption of telemedicine. One year of participatory design and analysis of the shared service centre concept involved stakeholders, such as clinicians, patients, technicians, policy makers, lawyers, economists and information technology architects. More than 100 people contributed to the findings. Most of the ideas generated for potential centre support for telemedicine could be categorised under four service categories. The need for such support services was verified in the cases investigated, and by agreement among stakeholders from regional health authorities, municipalities, and general practice. Therefore, it is probable that a shared service centre could help enable the wider deployment of telemedicine.

**Definitions:**

In this article, we use ‘telemedicine’ as an umbrella term for all the ‘tele-’ labels that are sometimes used rather indiscriminately to denote the use of information and technology to support healthcare services, including ‘telehealth’, ‘telemonitoring’, ‘telehomecare’, ‘e-health’, and so on. As per our definition, telemedicine may be synchronous and/or asynchronous, and may apply to any information and technology-based means of connecting healthcare actors and the patient, such as video communication, e-mail, electronic monitoring equipment, and Internet portals. Furthermore, the term ‘telemedical initiative’ covers projects in which telemedicine is conducted by a temporary project organisation, as well as self-contained telemedicine services used in daily, clinical practice in existing organisations.

## Introduction

For healthcare providers, telemedicine is increasingly seen as part of the solution to caring for ageing populations and the increased prevalence of chronic conditions.^1^ Interest in telemedicine has increased rapidly in recent years,^2^ and so has the debate about why so many telemedicine projects seem to be neither adopted in subsequent clinical practice, nor scaled up. One explanation for the elusive adoption may be that telemedical projects often deploy technology in an otherwise unchanged clinical practice, where clinicians in existing and unchanged settings manage telemedicine as an extra complexity on top of their daily clinical work.^3^ The Whole System Demonstrator (WSD) project,^4^ for instance, provided valuable lessons, but did not lead to whole system change, partly due to the randomised controlled trial (RCT) structure of the project, which required the organisations involved to develop service delivery in parallel with their other service deliveries.^4^ However, and not surprisingly, introducing digital communication does not itself imply substantial economic or clinical benefits.^5^ This may, in turn, at least partly explain why the majority of emerging pooled data reports and summative meta-studies (often favouring RCT studies in the inclusion criteria for their literature searches) find no hard evidence of improved clinical efficiency, compared to existing care,^6^ and the reason business cases generally favour traditional healthcare to telemedicine.^7^

The challenges of making telemedicine work after a project has ended seem to persist everywhere outside the strict scope of individual telemedical initiatives. Yet, most telemedical initiatives are evaluated presumptively on internal context and variables. Research examining personal experiences and process evaluations across different projects and organisational setups, informing processes of change and healthcare reform is sparse.^8,9^ Formative assessments of telemedicine are needed to complement summative reports.^10^ Hence, after more than three decades of experience with telemedicine, there is still no simple answer to essential questions about telemedicine, such as the following: How do health organisations embrace and adopt the changes in communication patterns,^11^ roles and responsibilities,^12^ personal relations,^13^ process coordination,^14^ and so on, which occur when healthcare services are carried out via telemedicine? Little attention has been paid to the levels of technical and organisational support needed to redesign, adapt, and translate existing practices along with the development of telemedical services.^1^

From a technological perspective, models for providing wider ‘Product Service Systems’^15^ or ‘Ecosystems’^16^ are emerging as steps beyond deploying telemedicine in ‘technological islands’, without integration into existing system and practices. Methodologically, theoretical frameworks for health information technology (IT) implementation and assessment are also beginning to appear.^17^ Contributing to this strand of research, this article has its starting point in results from a Public Private Partnership project for developing a new concept, a ‘shared service centre’ (SSC) for telemedicine. The purpose of the SSC project was to design an organisational construct that could work across different telemedical setups, and leverage the adoption and practice of telemedicine. The hypothesis underlying the project was that hospitals, municipalities, and general practitioners would be more motivated and able to adopt telemedical work in practice, if supported by the right service infrastructure. Furthermore, the telemedical work could be supported across different actors, and the SSC could offer patients improved access to their own data, thus supporting patient empowerment, and cooperation between primary and secondary healthcare sectors.

This article presents the results of 1 year of participatory design (action research) and analysis of the SSC concept, involving stakeholders, such as clinicians, patients, technicians, policy makers, lawyers, economists, and IT architects in the enquiry. The reason for choosing a participatory approach was that having a high level of user-group activity as the driver for the conceptualisation process created opportunities for engagement and dialogue with a broad range of stakeholders, whose subsequent support and ownership at strategic levels were prerequisites for eventual implementation of the concept. Thus, we regard the relatively dense process as important for engaging and aligning key persons at both operational and organisational/strategic levels. Creating a shared organisational vision is one step towards breaking the vicious cycle reported by May et al.,^18^ as a shared vision may counteract stakeholders’ ‘seeing each other as barriers to, not facilitators of, change’.

This article reports both the results of the conceptual development and the lessons learned in the process. Theoretically, its contribution may be regarded as a set of conceptual examples of issues that must be considered in the deployment of telemedical initiatives, and the SSC concept may inspire future research.

## Methods

Research-wise, action research (participatory design) has been utilised as the methodology,^19^ as qualitative enquiry framed by a series of project activities involving users. In various workshops throughout the project, participants were supported in grasping the complexity of the discussions through the use of various tangible approaches, where role-play, or the cooperative creation of various artefacts, was central to establishing common ground among the participants.

The work group (the authors) that conducted the bulk of the work on the project frequently called in a project group (15 participants) with representatives from two different municipalities, one healthcare administration region, one hospital, three private companies, one industry association, and one research institution. A series of workshops and meetings also involved a range of various potential users of an SSC, including clinicians (10 doctors, 20 nurses, and 5 other), representatives from private companies,^20^ public authorities (technical, legal, and financial – 29 in total), and healthcare organisation leaders.^11^ Patients (four) were included in interviews and field studies. Participants were recruited informally, using the various networks and projects of the municipalities and the healthcare administration region (convenience sampling), ensuring that both those who had questioned or criticised the concept, and those who had expressed positive opinions were invited to participate. In total, more than 100 persons contributed to the findings presented in this article through interviews/observations, workshop participation, and/or by responding to specific questions from the project group. The project activities are listed in Table 1.

**Table 1.** Overview of the project activities.

Idea generation phase, 3 months Activities:	- Interviews with project leaders and other participants from 12 different telemedical initiatives across the Central Denmark Region
	- Literature review/desk research
	- Two ‘Co-creation’ workshops, where telemedical work situations were elaborated by role-play in simulated/lab settings, with a focus on identifying challenges and generating ideas for potential support by an SSC (Figure 1(a))
	- Analysis and elaboration of an ‘idea catalogue’ for the SSC
Development phase, 5 months Activities:	- A ‘road show’ consisting of eight meetings, in which preliminary models and scenarios of the SSC were elaborated in a discussion with representatives from various perspectives, for example, health policy makers, clinicians from hospitals, municipalities and general practice, lawyers, economists and IT architects
	- A ‘materials’ workshop (Figure 1(b)), conceptualising the SSC model as a physical artefact, composed of bits and pieces of paper, wood and transparent layers of plastic
	- Four workshops in which the Osterwalder Business Model Canvas^21^ was used as a tool for conceptualising the value and role of an SSC in relation to cross-sector patient pathways
Conceptual test and evaluation phase, 4 months Activities:	- Three different telemedical initiatives were selected as test cases, and scenarios for the initiatives’ potential use of the SSC were elaborated in collaboration with their respective project crews, using a storyboard technique (Figure 1(c))
	- Organisational sketch workshops using a strategic board game (Figure 1(d))
	- Triangulation, and analysis of the findings according to technology, organisation, economics and law

SSC: shared service centre.

The purpose of the idea generation phase was to pinpoint and reflect on the challenges associated with a set of telemedical work situations from various, active telemedical projects, in order to generate ideas about the potential role of an SSC rooted in verified needs. Following the idea generation phase, the ‘idea catalogue’ covered more than 300 ideas for tasks to be performed by an SSC. The ‘road show’, the materials workshop, and the Business Model Canvas process served to condense, validate, and structure the findings. At the end of the development phase, the SSC concept had been elaborated and condensed into a general description of four, interdependent service categories that an SSC should facilitate, for telemedical initiatives to take full advantage of their support.

For the test phase, three different telemedical initiatives were selected to represent a variety of telemedical contexts, in order to span the greater part of the SSC idea catalogue. Unfortunately, none of the three projects has been published through international channels, which tends to be the case of the majority of Danish telemedical projects. The three initiatives and their telemedical services are briefly described here:

1. A project setup where a cross-sector telemedical service is established to handle heart insufficiency, chronic obstructive lung disease, diabetes, and elderly medical patients (Telemedical service: triage, and follow-up on patient self-monitoring and patient empowerment. Approximately 200 patients included.)
2. The national deployment of telemedical foot ulcer assessment (Telemedical service: specialist advice to visiting nurses performing wound treatment in the home of the patient, based on communication and sharing of digital wound images between primary and specialist actors. More than 10,000 patients included.)
3. A municipal project of virtual ‘exer-game’ rehabilitation following surgery (Telemedical service: interactive rehabilitation system providing feedback to the user on the accomplishment of the training programme, with the possibility of remote physiotherapist review and advice. In total, 20 patients included.)

**Figure 1.**
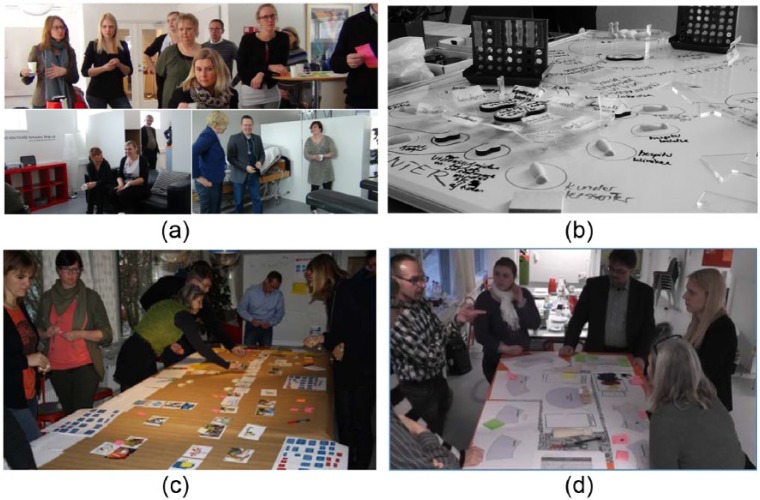
(a) Co-creation workshop, role-play, and reflection; (b) materials workshop and conceptual development; (c) storyboard workshop; and (d) strategic board game.

All three cases were elaborated using the storyboard method.^20^ The overarching purpose was to test whether the four service categories could be mapped onto the needs of the telemedical initiatives, but the workshops also gave rise to new ideas and development of the SSC concept. A storyboard consists of postcards arranged on a long table (see Figure 1(c)), depicting the various stakeholders, roles, and activities involved in a generic patient pathway in a telemedical initiative. First, a storyboard was made according to the current pathway of the projects. Second, the possibility of SSC support was added, and the storyboard was altered/co-designed, according to the new possibilities. By analysing the difference between the two storyboards, it was possible to evaluate the potential and challenges of using an SSC as a lever for optimising the work flow.

During the evaluation phase, there was also a focus on potential organisational implementation of the SSC. Since SSC span a multitude of skills and resources, it is unlikely that one organisation or one company alone could provide all relevant services. This means that SSC must be implemented through a bricolage of actors, each offering elements of the services and functionalities, but within an overarching structure, to ensure coherence and cooperation. To take an initial stab at formulating recommendations for this complicated issue, a strategic board game was developed to test and evaluate various organisational sketches, by discussing how, in the different sketched implementations; the SSC might tackle different situations from the test cases/storyboard scenarios.

The material originating from the various activities (video recordings and transcripts of workshops, field notes, meeting minutes, co-produced material, such as posters and drawings) was analysed by the work group and presented to the project group approximately once a month. This served as a way to iteratively capture and drive forward the common understanding of the findings. Before writing this article, the work group revisited all material generated during the project (more than 200 distinct files, not counting photos) and made triangulations according to themes, including technology, organisation, economics, and law. Thus, the majority of findings and lessons learned presented below are the overall results of the project rather than products of using one specific method.

## Results

The envisioned SSC concept emerging from the development efforts may be described as a virtual organisation enabling the various stakeholders (patients, hospitals, municipalities, general practitioners, and private companies) to share resources and act together in work related to the (telemedical) course of treatment for every patient. The SSC does not constitute a new healthcare authority on its own, but it makes it possible to shift and share responsibilities and resources among the stakeholders.

A requested scheme is created at the SSC for each patient, comprising the list of tasks to be performed, providing the stakeholders with a central means of coordinating clinical, technical, and economic aspects across the boundaries between healthcare sectors and companies. Our preconception of the need for a concept such as this was strengthened by the investigation of a range of local/regional telemedical initiatives conducted in the idea generation phase (see Table 1). Aligning with international experience, the telemedical initiatives investigated in the Central Denmark Region were mainly supported by enthusiastic project groups or management from a specific department. No dedicated telemedicine support role was appointed to make the telemedicine service work within current clinical practice. The projects included up to as many patients as could be handled by the project clinician (a rule of thumb seems to be around 100 active telemedical patients per nurse), and none of the projects was scaled beyond this threshold.

### Four categories of services

During the development phase, four general service categories were defined, comprising the majority of the generated ideas for tasks to be performed by an SSC. The categories were appropriately mapped onto the needs of the three telemedical cases used in the storyboard work, and we consider the categories important for encapsulating the project visions. The service categories are presented next.

#### Technical support and logistics

In current telemedical projects, healthcare professionals and ‘project people’ spend much time on technical and logistical tasks associated with telemedicine. The SSC must provide first-level technical support to healthcare professionals and patients, delivery and pickup of telemedical equipment, setup, installation and testing, system administration, cleaning and preparation of equipment for other patients, and educating users in its use. The SSC does not dictate specific solutions, but would support a range of certified products.

#### Information and coordination

SSC must run a platform for data sharing, which will make it possible to obtain an overview of the contacts with various healthcare providers, the data collected by the telemedical solution, and the patient’s consent to data sharing and participation in telemedicine. For this to work, the SSC must offer a set of open interoperability standards and facilitate a certification process, where third-party telemedical solutions may be integrated with the SSC platform. The important aspect is the support of cross-sector communication and coordination, to support a shared healthcare network acting for the patient.

#### Self-service and personal care coordinator

Extending the information and coordination system, a web interface must provide the patient with access to calendars, contact data, monitoring data, reports, communities of peers, and so on, in order for them to take control of healthcare coordination. If neither patient nor any relative is capable of arranging this coordination, a healthcare professional (from a hospital, municipality, general practice, or the SSC) could be commissioned to assume the role of care coordinator. In some telemedical initiatives, well-defined clinical tasks could also be assigned to the SSC, for instance, sorting and annotating data from monitoring equipment, on the basis of set algorithms.

#### Knowledge and development centre

To anchor telemedical initiatives in best practice, both clinically and organisationally, the SSC must facilitate a knowledge centre that collects experiences and resources related to various, certified telemedical solutions. SSC can also guide development processes of systems intended to be included in the portfolio of certified solutions. Finally, SSC should use the evidence base to help inform health strategies and the development of best telemedical practices.

### Creating value with the SSC

All three telemedical cases used in the storyboard workshops verified the need for the four service categories and, in particular, the interdependence of these. As an illustration of this, the process of including a patient in a telemedical initiative involves assessment of both technical and clinical issues, coordination of a patient’s technical and clinical education, coordination of technical installation, initiating the clinical protocol, as well as obtaining informed consent for data collection and sharing. The various services may be provided by different actors or companies, but the various actions should be closely coordinated, or even combined in a virtual setup, to provide a smooth and efficient patient pathway. Therefore, the four categories should be seen as a whole.

The storyboards showed that the collection of administrative and business-related data, such as key performance indicators to be used by the knowledge and development centre, should be seen in connection with the other categories, as often, they may be handled during the same work procedures that document technical enquiries and clinical communication with the patients.

During one workshop, a discussion elaborated the recurring issue of whether the specialist clinicians at the hospital could outsource the responsibility of monitoring daily measuring data to an SSC without compromising patient safety. One specialist nurse who controls a telemedical initiative considered this and concluded that ‘If I don’t need to view the daily data, then I could just as well discharge the patient to general practice’. That gave rise to the hypothesis that if the SSC ensures and coordinates the proper level of advice from specialists, then primary care physicians may assume responsibility for some specialised telemedical courses of treatment, which may be a more cost-effective model than placing that responsibility with secondary care institutions.

Generally, the point of view that emerged in the project group during the development phase and conceptual test was that the four service categories have the potential to support healthcare professionals in the municipality, hospitals, and general practice, to focus on their core tasks, and not spend time on coordination, logistics, training, technical support, and ‘trivial’ clinical tasks, such as sorting data. The SSC may perform these tasks more efficiently on a large scale than could individual actors, peripherally.

Finally, SSC may ensure continuity of the individual patient’s courses of treatment, and facilitate cross-sector coordination of data and services in the pursuit of a more holistic approach to patient care. By providing patients or their relatives with access to their data and treatment plans, the SSC could, potentially, leverage patient empowerment and co-determination in the courses of treatment.

### Concerning technology, organisation, economics, and law

#### Technology

Current telemedical initiatives often involve the parallel use of dedicated IT systems and existing IT systems in the healthcare sector. The lifecycles of these systems often intertwine with the launch and conclusion of development/pilot projects deploying the telemedical services, and the telemedical solutions or their purchasers may not demand compliance with open integration standards.

**Figure 2.**
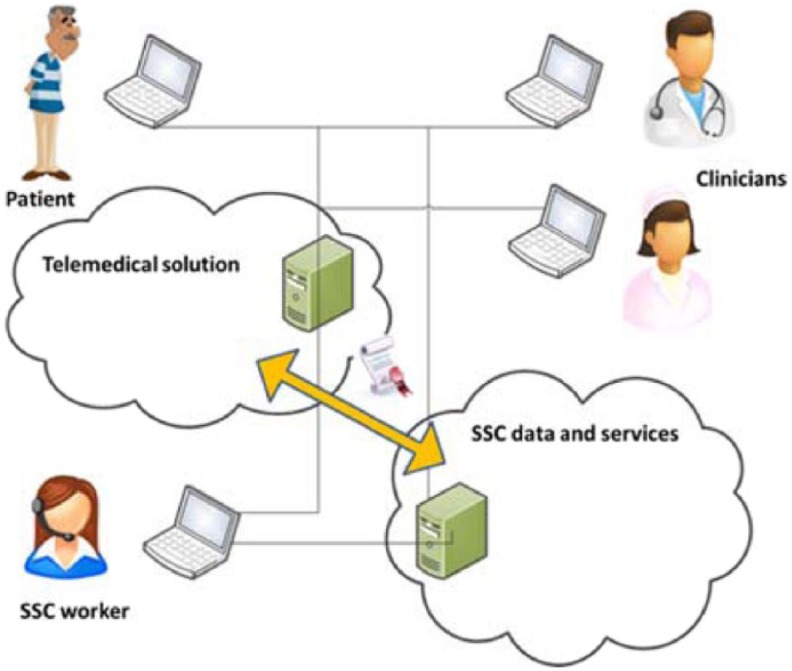
SSC services list requirements of, and certify, telemedical services.

‘Road show’ meetings, technical discussions of the development workshops, and input from IT architects from the regional authorities concluded that the SSC needs to be established on a set of open standards to ensure interoperability among different telemedicine solutions, and compatibility with the existing healthcare IT infrastructure, such as the electronic health records at hospitals and in municipalities. The Danish national infrastructure utilising international interoperability standards (in particular, from Integrating the Healthcare Enterprise (IHE), health level 7 (HL7), and Continua Health Alliance) is in the process of addressing part of the integration challenge, but progress is slow, and interim solutions may be necessary to bridge the gap. Thus, in order to implement an SSC, it would be necessary to define the interface among the various and changing telemedicine systems, and a persistent backbone SSC system, as shown in Figure 2.

We conclude that the implementation of an SSC will assign requirements to the IT systems deployed in a telemedical initiative, either in the form of a standard/certificate that ensures interoperability, or by requiring the telemedical solution implementation to make use of interface components within the SSC system. Either way, changes in the underlying deployment of telemedical initiatives are inevitable, which in turn challenges the adoption of telemedical cases, as already deployed initiatives will be resistant to changing their technical setup.

#### Organisation

The project group included both public and private companies. Public–private cooperation is seen as a key requirement for deploying an SSC that takes into consideration both the complexities of cross-sector healthcare services delivery and optimises work flow. Although the private companies in the consortium were relatively aligned in their position towards the projected setup, different opinions prevailed among the various public authorities, in particular, between the regional health authorities with their focus on hospitals, and the municipalities, focused on primary healthcare.

A recurring question during the ‘road show’ meetings (see Table 1) was who should own the SSC. Reaching a model that was generally agreed upon by the different stakeholders became problematic. Representatives from municipal institutions argued that the greatest incentives for utilising telemedicine were to be found in the primary healthcare sector and the municipalities. The general practitioners emphasised their potential role in the SSC as personal care coordinator, and representatives from Central Denmark Region frequently said that the regional authorities would have the necessary resources and capabilities to run the SSC. Although all participants were eager to position themselves to ‘get the order’, they were more hesitant about working with one another’s services.

The discussions during the board game workshops (see Figure 1(d)) captured some of these principal difficulties. Here, the evaluations of the different sketches for potential organisational implementation revealed that if the SSC was not rooted in shared ownership across municipalities and regions, parallel structures would quickly materialise between the different public actors, thus preventing the harvest of the synergetic benefits that SSC was intended to produce in the first place. Shared ownership between municipalities and regions seems to be the prerequisite for a way forward, to create engagement with the implementation on both sides.

Regardless of ownership, the participants in the board game workshops (see Figure 1(d)) found it important that the SSC allows all stakeholders to participate simultaneously in both delivery and use of SSC services. In particular, SSC should not constitute a new healthcare sector, and therefore, clinical work performed under the SSC umbrella should be performed by clinicians employed elsewhere. This could be done by arrangements similar to the job rotation schemes in Yorkshire and the Humber, where manning the service desk was typically considered a part-time occupation.^22^ Finally, it is important to organise the SSC in a way that enables patients to take an active role in the telemedical course of treatment.

#### Economics and law

In principle, the business model of the SSC should simply be that the SSC gives good value for the cost of using the service. The various telemedical initiatives making use of the SSC services could be regarded as the customers. However, the Osterwalder Business Model Canvas workshops (see Table 1) and the board games revealed that this business model suffers from some weaknesses.

When deciding to launch a telemedical initiative, projects often focus on internal aspects, such as the clinical effectiveness of an intervention, or cost effectiveness of a specific work flow. In contrast, most of the identified potential of the SSC may be characterised as external aspects, seen in relation to the individual telemedical initiative, as they create value on more general levels, such as scalability of services, cross-sector cooperation, and continuity. Furthermore, these aspects are difficult to evaluate and compare to the existing organisation, due to lack of available baseline measures. Public healthcare management has surprisingly little data about the actual cost of telemedicine. For instance, the time spent on telemedical coordination work is usually invisible in the running accounts, as telemedical initiatives seldom measure such data explicitly.^23^

The underlying cash flow for an operational SSC cannot be based solely on a ‘fee for service’ model, where the SSC is financed by the participating telemedical initiatives only. A current task of the ongoing SSC project is to identify a suitable framework that combines an array of financial channels and cash flows, such as public procurement, overhead fees on services, co-financing by contractors and health insurance companies.

Concerning legal aspects, the medical liability for a cross-sector course of telemedical treatment, with the active participation of the patients themselves, needs clarification. Although we do not see SSC taking the overarching clinical responsibility (i.e. we do not want to invent a new sector), some tasks performed by SSC, such as data filtering, may be regarded a ‘grey’ zone, in terms of liability.

### Resistance to change

As is evident from the preceding sections, the discussions during the project were heavily affected by resistance to change, which is typical of the healthcare domain.^3^

Most participants could see the advantages of delegating technical tasks and logistics to external providers and, regardless of their profession and occupation, many could also see the potential benefits of delegating certain clinicians’ tasks to a service centre. However, some of the participating clinicians, in particular, simultaneously felt a resistance to conceding these tasks.

At an early workshop, one nurse argued that in the telemedical initiative in which she had a predominant role, one of the things that made it all work was that she was involved in the entire communication regarding the patients, and thus had a very personal relationship with, and detailed knowledge of every single one. Delegating some of the work to a service centre would, arguably, insert an extra link in the chain between her and the patient. However, she also acknowledged that the current setup was not very scalable, since she would not be able to handle more patients than she already did. That prompted this remark: ‘We have designed this [a local telemedical initiative aimed at medical treatment of heart insufficiency], based on the evidence of best clinical practice. Now you ask me to think out of the box. It is not easy’.

At strategic levels, a certain amount of resistance was also encountered. When designing the test phase for the project, the original intention was to make a more elaborate test with a limited, prototypical organisation. However, the message from hospital management was that during the test ‘no one must be harmed’. Although a perfectly understandable requirement, it also demonstrates the inertia in the healthcare sector. Another potential deal-breaker was if persons involved at the strategic level identified the project ideas as ‘service extensions’. Particularly among representatives from municipalities, even mentioning service extensions is completely unacceptable, as the ideas then conflict with the ongoing effort to keep down public expectations of the service level. The conclusion is that although an SSC may support a range of tasks, to make telemedicine more scalable, the successful implementation of an SSC will depend on the willingness of a range of people to do things in a different way.

## Discussion

The SSC project set out to identify a better way to integrate telemedicine into healthcare, not by inventing a new healthcare authority, but by pursuing a virtual organisation combining the existing resources. Did we succeed? We do not know. This article marks the end of the first cycle of conceptual development and evaluation, but the project is not yet completed. The public authorities funding the first stage of the project have asked for a continued effort, and currently, a test is being prepared to implement and test parts of the concept in practice, in cooperation with existing telemedical initiatives. In parallel with this test, an organisational process has been launched to design and implement the legal and economic base for further deployment of the SSC concept.

The three test cases all verified the need for the suggested services of the SSC. By involving more than 100 stakeholders, each of whom could see the point, and some of the potential of the SSC concept, the project has, without doubt, left fingerprints on the various telemedical efforts and developments in this area. We consider the establishment of a common ground between regional health authorities, municipalities, and general practice an important accomplishment. Furthermore, the SSC concept could be used analytically and constructively, to discuss current barriers and potential regarding current telemedicine initiatives.

We knew that ours was an ambitious project, and significant resistance to change was expected. It is clear that a conceptual project does not affect culture or trigger a paradigm change on its own. However, the project may serve as a common reference point for the various stakeholders in the project consortia, and identify the various issues and challenges at stake. We hope that in doing so, the project has helped prepare the ground for further change, and the integration of telemedicine in practice.

Theoretically, we believe our findings may also make a more general contribution. Several previous telemedical projects have implemented telemedicine centres, where dedicated, technical and clinical staff mans the telephone lines, performs triage, or makes preliminary diagnoses based on telemonitoring data. Often, these centres are organised as an extension of the manufacturer of the specific telemedical solution, focusing on the specific protocols deployed in a telemedical initiative. For example, Müller et al.^24,25^ report on a range of studies of chronic heart failure patients, where the daily data from the monitoring equipment in the patients’ homes were collected and triaged by a telemedical service centre. Emergencies could be handled thanks to the round-the-clock presence of clinicians at the centre. Although these and other examples touch upon some of the same issues addressed in the SSC project, the generic perspective of SSC has not yet been proved in real settings. For example, most previously reported service centres seem to depend on the concrete telemedical project/service – that is, heart monitoring centres for heart monitoring projects, or services specific to a geographical area. Yorkshire & the Humber Telehealth Hub^22^ tried to reuse telemedicine centre services across three different areas within the region, but uptake of the services outside their own areas was low, and the intent to have single contacts across all three areas proved too complex in practice.

In the end, we do not know whether the complexity will also be too big a challenge when it comes to SSC, but by identifying four generic service categories that could be developed and governed generically, the SSC concept may inspire future theories about the implementation and adoption of telemedicine. The validation of the storyboard concepts for three different telemedical initiatives and the consensus among the various stakeholders supports this belief.

A limitation of this study is the limited degree of patient involvement. Patients were included in interviews, but most of the workshops provided a space for co-design discussions of complex issues of technology and organisations among professional users, and inevitably (not intentionally), the main focus was on medical staff and supporting clinical work, so that the SSC, in turn, could support telemedical initiatives in paying more attention to the needs of the patient. Future work should elaborate the patient perspective of the SSC concept, for instance, by designing support for the daily ‘bricolage’^26^ of devices and services, to make use of and make sense of telemedicine.

The lack of coherence among healthcare sectors, the limited opportunity for patient empowerment, the difficulty of fitting chronicity paradigms into current healthcare organisations, and so forth, will not go away any time soon. Telemedicine holds a key for general healthcare reform. We consider it probable that an SSC offering the services described above would be likely to lower the barriers to adoption of telemedicine and changed, clinical telemedical practices, but the road ahead is long and winding.
